# Editorial: Suicide and self harm in young people

**DOI:** 10.3389/fpsyt.2022.1120396

**Published:** 2023-01-10

**Authors:** Elaine M. McMahon, Laura Hemming, Jo Robinson, Eve Griffin

**Affiliations:** ^1^School of Public Health, University College Cork, Cork, Ireland; ^2^National Suicide Research Foundation, Cork, Ireland; ^3^Centre for Mental Health Research, National Centre for Epidemiology and Population Health, Australian National University, Canberra, ACT, Australia; ^4^Orygen, Melbourne, VIC, Australia; ^5^Centre for Youth Mental Health, University of Melbourne, Melbourne, VIC, Australia

**Keywords:** suicide, self-harm, prevalence, service utilization, young people

## Introduction

Suicide is a leading cause of death among young people worldwide, and a history of prior self-harm is the strongest predictor of subsequent suicide (1, 2). Community-based studies have shown that ~10% of young people have harmed themselves (3), with recent research suggesting an increase in the incidence of self-harm and suicide among this group, especially in children (4, 5). Internationally, young people are facing what has been described as a “rising tide of mental ill-health” (6). Increasing rates of mental disorders have been reported, including anxiety and depressive symptoms and, in some regions, suicide-related outcomes (7–11). Self-harm in young people remains, for the most part, hidden within the community, and so providing timely and targeted interventions remains a key challenge for clinicians and for those working in the broader area of youth mental health. This Research Topic aimed to add to our understanding of the factors underlying suicidal behavior in adolescence, global trends in the incidence of youth self-harm and suicide, and potential mechanisms and modifiable factors which may reduce risk of self-harm and suicide in young people. The 17 published articles broadly represent themes of risk factors and prevalence, service utilization and experiences of suicide and self-harm.

## Prevalence of suicidal behavior and associated risk factors

Several studies published in this Research Topic have examined the prevalence of non-suicidal self-injury (NSSI) among young people, in a variety of populations. Xiao et al. carried out a meta-analysis of studies involving 264,638 adolescents to estimate the prevalence of NSSI in non-clinical adolescent populations. The authors reported an aggregate prevalence of past-year history of 23.2%. A study carried out in Israel by Hamdan et al. reported higher rates of NSSI than the aggregate prevalence reported by Xiao et al., with 30.7% of the sample reporting that they had engaged in NSSI. In this study, elevated rates of NSSI were found in males, those from an immigrant group or Muslim minority, those with severe depressive symptoms and internet addiction. A study by Huang et al. which surveyed Chinese adolescents found that 33.9% of the participants had engaged in NSSI in the past year. Childhood trauma and psychological symptoms increased the risk of NSSI by two and five times, respectively, while high levels of psychological symptoms were found to have partial mediating effects between childhood trauma and NSSI.

In a study from Morocco (Tom and Mafoud), Global Schools Health Survey (GSHS) data in relation to suicidal ideation and planning were analyzed. GSHS is an international collaborative surveillance project designed to help countries measure and assess the behavioral risk factors and protective factors in key areas among young people. Suicidal ideation was reported by 14.4% and suicidal planning by 12.9%. Suicidal ideation was found to be associated with identifying as female, increasing age, bullying victimization, feeling lonely, cigarette smoking, marijuana use, and hunger frequency. Planning was associated with a lower educational level and living in a rural area.

In a school-based survey by Xu et al., conducted in three provinces in China, associations between psychological symptoms, suicide attempts (SA), and NSSI were examined in young people aged 10–20 years. Psychological symptoms and NSSI were independently associated with a higher likelihood of suicide. Adolescent boys with psychological, conduct or social adaptation symptoms without concurrent NSSI were almost three times more likely to report SA than those who reported NSSI, while in girls, only those with social adaptation symptoms had a higher risk of SA in the non-NSSI group than NSSI group.

Several studies examined novel methodologies or emerging risk factors for suicidal behavior in young people. The importance of biological markers were explored in work reported by both Abrial et al. and Barzialy et al. Abrial et al. present the protocol for a novel prospective study to assess the risk of re-attempting suicide and to investigate the multidimensional predictive factors associated with re-attempting suicide in youth after a first suicide attempt. Several socio-demographic, clinical and biological assessments will be undertaken in this promising work (Abrial et al.). Barzilay et al. evaluated the contribution of a polygenic risk score for suicide attempt (PRS-SA) in explaining variance in suicide attempt by early adolescence and conclude that PRS-SA may be useful for youth suicide risk classification.

Experience Sampling Methods (ESM)—which collect self-report information on experiences, emotions or behaviours from an individual as they occur in-the-moment—were explored in two studies. Findings of a study by Kirtley et al. indicate that short-term future thinking relates to suicidal ideation among a non-clinical sample of adolescents. Participants reporting higher past-week suicidal ideation reporting significantly less daily positive future thinking, suggesting a potential role in the development of suicidal thoughts and behaviors. Williams et al. report on the feasibility and acceptability of ESM among LGBTQ+ young people with self-harm thoughts and behaviors, finding that such methods are both acceptable to young people and feasible. The authors stress the need for a full-scale study to better understand temporal trends within this population.

In a timely systematic review, Scudder et al. sought to identify and describe empirically tested screening tools for suicidality in youth presenting to Emergency Departments (ED). In the included studies, the most researched tools were the Ask-Suicide Screening Questions (ASQ) (*n* = 15), Columbia-Suicide Severity Rating Scale (C-SSRS) (*n* = 12), Suicidal Ideation Questionnaire (SIQ) (*n* = 11), and the Risk of Suicide Questionnaire (RSQ) (*n* = 7). Where screening was applied to all patients, about one-fifth of pediatric ED patients screened positive; where suicide screening was applied to psychiatric patients only, over half screened positive. The authors suggest that such screening tools may help to support early detection and appropriate intervention for youth at risk of suicide.

## Patterns of service utilization among young people

A number of studies examined help-seeking behavior and service utilization in young people prior to suicide or as a result of self-harm or suicidal ideation. Geulayov et al. examined the utilization of formal, informal and online supports accessed by adolescents before and during the first lockdown period of the COVID-19 pandemic in England. Approximately 13% of adolescents surveyed reported having ever self-harmed, and 7% reported to have self-harmed during the lockdown period. Help-seeking following self-harm was low, with more than one-third of young people not receiving any help. Most commonly, adolescents reported accessing support from friends, with few accessing formal or online supports. Common reasons for not accessing formal supports reflected stigma. The authors suggest that identifying ways to mitigate barriers to help-seeking as well as improving the perceived helpfulness of supports is warranted.

Two studies from the United Kingdom provide interesting overviews of the profile of young people experiencing suicidality—highlighting parental separation or loss, bullying, and autism spectrum disorder (or the presence of autistic traits) as important contributory factors. Both studies highlight the importance of accurate coding of such presentations by clinical services in order to accurately represent the number of presentations and subsequent referral pathways. Ashworth et al. presented a case series study of emergency department presentations by children and young people, which identified an increase in such presentations during the COVID-19 pandemic. Many were currently engaged with or referred to Child and Adolescent Mental Health Services (CAMHS), a finding reflected in a study of Scottish referrals (Gilmour et al.) of CAMHS referrals, where 24% of all referrals were for suicidality. Their study further demonstrates the clear need for specialist self-harm teams within CAMHS in order to provide appropriate assessment and management of such presentations. They also highlight the need for early intervention in children under the age of 12 years, who are less likely to be referred for intervention.

Service utilization *via* CAMHS was further explored by Astrup et al., who examined service utilization in the year prior to suicide in a cohort of Norweigan young people. One-quarter of young people who died by suicide had contact with CAMHS in the year prior to death. Boys were less likely to have had contact with mental health services in the year prior to death and were four times more likely to have terminated contact at the time of death. The authors discuss the importance of strategies to improve service contact for boys in particular, as well as the need for more universal programs to address mental health of young people.

## Young peoples' experiences of suicidal behavior

Finally, a number of studies in this Research Topic have utilized a range of qualitative methodologies to explore young peoples' experiences of suicide and self-harm. In Canada, Harding et al. conducted semi-structured interviews with caregivers of children and youth with fetal alcohol spectrum disorder with a view to exploring their perceptions of the young person's suicidal experiences. They used interpretative phenomenological analysis to form a composite vignette which depicted a single all-encompassing narrative organized around the social-ecological suicide prevention model. This comprised individual level factors such as sociodemographic characteristics, co-occurring health conditions, substance use, early life trauma and familial conflict. Secondly, relational factors such as feelings of belonging (or lack thereof), social disconnection, bullying, and the influence of peer groups were identified. Thirdly, there were a number of community level influences of suicidality centering on regions or settings such as neighborhoods, schools, workplaces, and interactions with healthcare systems. Finally, societal level factors were emphasized including issues such as stigma, geographic region (urban vs, rural settings), and the impacts of the COVID-19 pandemic on experiences. The authors state that their findings suggest the need for training and advocacy to ensure that mental health systems can appropriately respond to the needs of young people with fetal alcohol spectrum disorder experiencing suicidality.

In New Zealand, Van Wyk and Gibson conducted a thematic analysis on pre-existing transcripts of text communications between young people and a counseling helpline. Young people expressed that suicidal thoughts were a part of everyday life for them and they viewed suicide as an escape from their reality. However, young people also stated that they were ambivalent about dying. Young people also stated that they used suicidality to convey their anguish and connect with others. Young people spoke about the varying intensity of their suicidality, including when their thoughts were perceived to be out of their control. Around half of the young people stated they had made a plan for suicide. A number of young people recognized their need for help and support in regards to their suicidality. This study provides a novel insight into how young people themselves communicate their experiences of suicide in real-time.

In the United Kingdom, Norman et al. conducted semi-structured interviews with young women about their experiences of self-harm. Four themes were discovered *via* an interpretive phenomenological analysis, though just one of these is discussed in the brief report published in this Research Topic; “Is self-harm bad?”. In this study, participants both acknowledged and resisted the social construct of self-harm as “bad”. In particular, they resisted the idea of self-harm being “bad” due to beliefs that self-harm: was a symptom of underlying mental health difficulties or life stresses; “worked” for them as a coping mechanism; was a part of their identity and narrative. These findings provide additional insights into the way in which people who self-harm navigate the prevailing perceptions of the behavior. Such insights are a crucial step toward the goal of reducing recurrence of self-harm.

## Conclusion

Through this Research Topic we sought to better understand the factors underlying suicidal behavior in adolescence, global trends in the incidence of youth self-harm and suicide, and innovative interventions to reduce self-harm and prevent suicide among young people. The body of research represented in this collection highlights novel methodologies and the importance of qualitative research when understanding how best to support young people. A key aim of this Research Topic was to have good representation of studies from low-middle income countries (LMICs), where a significant proportion of youth suicides occur, and from hard-to-reach groups (12). While many articles continue to be from high-income countries (HICs), we are seeing emerging research on ethnic minorities and groups who may be at increased risk of suicide. Future research priorities in this area include the involvement of those with lived experience in youth mental health research, the perspectives of caregivers and families, and the need for high-quality intervention studies.

## Author contributions

EM, LH, JR, and EG contributed to the editorial and approved the final version.
